# Effects of the in ovo injection of an *Escherichia coli* vaccine on the hatchability and quality characteristics of commercial layer hatchlings

**DOI:** 10.1016/j.psj.2023.103057

**Published:** 2023-08-22

**Authors:** S.A. Fatemi, L.L. Lindsey, J.D. Evans, K.E.C. Elliott, S.A. Leigh, K.J. Robinson, A. Mousstaaid, P.D. Gerard, E.D. Peebles

**Affiliations:** ⁎ Department of Poultry Science, Mississippi State University, Mississippi State, MS 39762, USA; † USDA-ARS, Poultry Research Unit, Mississippi State, MS 39762, USA; ‡ School of Mathematical and Statistical Sciences, Clemson University, Clemson, SC 29634, USA

**Keywords:** chick, *E. coli*, hatchability, in ovo, layer

## Abstract

In the commercial egg industry, avian pathogenic *Escherichia coli* (**APEC**) can lead to significant economic loss. The Poulvac *E. coli* vaccine (**PECV**) is a commercially available attenuated live vaccine commonly applied via spray or drinking water to protect against losses associated with colibacillosis. The PECV has not been tested in layer hatching eggs using in ovo injection. Therefore, the purpose of this experiment was to determine the effects of injecting 50 μL of different doses of the PECV into Hy-Line W-36-layer hatching eggs on the hatchability and quality characteristics of hatchlings. At 18 d of incubation (**DOI**), treatments included 1 noninjected and 1 diluent-injected control. Furthermore, PECV treatments included a full dose (4.4 × 10^8^*E. coli* CFU) or serial dilutions of the full dose to produce 4.4 × 10^6^, 4.4 × 10^4^, or 4.4 × 10^2^ CFU doses of *E. coli*. In ovo injections targeted the amnion. Percent hatchability of live embryonated eggs (**HI**), percent residue eggs, hatchling mortality, and female chick whole and yolk-free BW, relative yolk sac weight, and body length were among the variables examined. Treatment significantly (*P* < 0.0001) affected HI, with HI being highest in the control groups (97.3% in the noninjected and 94.2% in the diluent-injected), and with HI values being 89.0, 88.9, 84.4, and 71.2% in the 4.4 × 10^2^, 4.4 × 10^4^, 4.4 × 10^6^, and 4.4 × 10^8^ CFU *E. coli* dose treatments, respectively. The percentage of live embryos that did not complete hatch but that pipped internally (*P* = 0.024) or externally (*P* < 0.0001) were significantly affected by treatment, with percentages being highest in the 4.4 × 10^8^ CFU treatment. Female chick body length was significantly (*P* < 0.0001) affected by treatment and was longer in both control groups and in the 1 × 10^2^ CFU *E. coli* treatment in comparison to all other treatments. Yolk-free female chick BW was significantly (*P* = 0.034) affected by treatment and was lower in the 4.4 × 10^6^ CFU and 4.4 × 10^8^ CFU treatments when compared to the diluent-injected control group. An increase in the *E. coli* concentration administered in the amnion of embryonated layer hatching eggs at 18 DOI decreased hatch success and female chick yolk-free BW and body length.

## INTRODUCTION

Avian pathogenic *Escherichia coli* (**APEC**) is a pathogenic strain of *E. coli* that can cause significant economic losses within the table egg industry (Barnes et al., 2008). Disease transmission and incidence can increase if birds are housed in undesirable conditions in which they are exposed to contaminated feces, are provided contaminated water, or are subjected to insufficient ventilation and high air humidity (Dho-Moulin and Fairbrother, 1999; Barnes et al., 2008). Infection in a layer facility can further be spread by the inhalation of dust particles (Dho-Moulin and Fairbrother, 1999). An infection of APEC can manifest itself in multiple ways, including yolk sac infection, cellulitis, swollen-head syndrome, salpingitis, acute fatal septicemia, peritonitis, airsacculitis, and orchitis (Barnes et al., 2008). Affected birds will normally show clinical signs such as the inability to eat and drink and can exhibit complications with their balance and gait (Barnes et al., 2008).

Early APEC infections can originate and spread in hatcheries, especially if floor eggs are used, and can subsequently lead to poor chick quality (Dho-Moulin and Fairbrother, 1999; Barnes et al., 2008). Avian pathogenic *E. coli* is one of the leading causes of first week mortality of chickens (Swelum et al., 2021). Due to this, the Poulvac *E. coli* vaccine (**PECV**; Zoetis, Durham, NC) was developed, and is the only modified live *E. coli* vaccine available to protect and improve the health of all major types of commercial poultry in the United States (Zoetis, 2021a,b,c). In commercial layer operations, it is usually administered to pullets through drinking water or coarse spray at 1 d of age and again at 14 to 16 wk of age (Zoetis, 2021a). Upon testing the effects of the PECV in laying hens at 5 d of age, and again at 14 and 30 wk of age, Christensen and Nielsen (2020) reported that it was safe and had no negative effects on egg production. Śmialek et al. (2020) have also shown that the PECV can minimize subclinical *E. coli* infection incidences and improve the production results of broilers.

The process of injecting a hatching egg with a substance intended to confer a benefit to the embryo and subsequent hatchling is known as in ovo injection (Peebles, 2018). This process usually takes place before incubation is completed and has been determined to be a safe and effective process, with little to no effect on chick hatchability (Peebles, 2018). In addition, to maximize the profitability of in ovo injection it is recommended that injections be given at 18 d of incubation (**DOI**) and that they target the amnion (**AM**; Williams and Hopkins, 2011). Utilizing in ovo injection can reduce the risk of bird stress, lower labor costs, and give reassurance that an equal dose is being distributed to each chick vaccinated (Ricks et al., 1999). In ovo injection of vaccines for other ailments besides APEC, such as those for coccidiosis or IBDV, have been found to confer immune protection when birds were challenged later in life (Moura et al., 2007; Sokale et al., 2018).

In a previous study conducted by Lindsey et al. (2022), it was shown that although the in ovo vaccination of 6.5 × 10^3^ and 6.5 × 10^4^ CFU of PECV provided a 100% level of *E. coli* presence in the AM, those levels led to significantly higher embryonic mortalities and a subsequently lower percentage hatchability of eggs that contained live embryos at the time of injection when it was administrated in the AM rather than air cell. For a better understanding of the effects of the in ovo administration of the PECV in commercial layers, in addition to a comprehensive determination of the effects of different doses of the PECV on the hatching process, a more extensive subsequent evaluation of the various quality characteristics of hatchlings is necessary. Therefore, the first objective of this study was to analyze the hatchery residue and hatchability percentage of Hy-Line W-36-layer embryos when vaccinated in ovo with various doses of the PECV at 18 DOI. A second objective was to determine the effects of these treatments on the subsequent quality characteristics of the hatchlings including yolk-free BW (**YFBW**), relative yolk sac weight, and body length.

## MATERIALS AND METHODS

### Egg Incubation and Treatment Assignments

All handling and care of the layer hatchlings were conducted under the approval of the Mississippi State University Institutional Animal Care and Use Committee (Protocol # IACUC-20-351). At 0 DOI, 3,240 Hy-Line W-36 (Hy-Line International, 2020) layer hatching eggs from a 46-wk-old parent flock were randomly allocated to 3 single-stage NatureForm NMC 1080 incubators (NatureForm Hatchery Systems, Jacksonville, FL). The parent flock had been vaccinated for *E. coli* based on the recommendations of the commercial source (Hy-Line North America, Mansfield, GA). The incubators served as both the setter and hatcher units for the study. Settable eggs were preassigned to 6 treatment groups, with treatments assigned to incubation trays containing a total of 90 eggs designated for an individual treatment. Two replicate blocks of incubation trays having a similar random arrangement of all 6 treatments were represented in each of the 3 incubators. Therefore, there were a total of 6 replicate groups (blocks) for each treatment. Eggs were incubated at standard set temperatures (37.5°C dry bulb and 29.8°C wet bulb temperatures between 0 and 18 DOI, and 36.9°C dry bulb and 29.3°C wet bulb temperatures between 18 and 22 DOI). Eggs were turned once every 2 h. At 12 and 18 DOI, eggs were candled to verify live embryonation. Nonembryonated eggs and eggs with dead embryos were discarded. Mean percent egg weight loss (**PEWL**) for each treatment-replicate group of eggs was calculated between 0 and 12 DOI and between 12 and 18 DOI. This was determined to establish the lack of an incubational vertical stratification effect on the arrangement of the treatment groups. All chicks were pulled from the hatcher at 22 DOI (hatch).

### Vaccine Preparation and In Ovo Injection at 18 DOI

All serial dilutions were performed in sterile Marek's disease commercial diluent (Merial, Inc., Athens, GA), and all titer determinations were determined from duplicate plates and were performed as described by Lindsey et al. (2022). Upon plating the full dose of the PECV, pre- and postinjection titers (CFU/mL) were observed to be 8.8 × 10^9^ and 3.4 × 10^9^, respectively. The full dose of the PECV was diluted using 10-fold serial dilutions to create 1 × 10^−4^, 1 × 10^−5^, 1 × 10^−6^, and 1 × 10^−7^ dilutions. In correspondence with the serial dilutions, the amount of *E. coli* delivered in a 50 μL volume to each egg were 4.4 × 10^8^, 4.4 × 10^6^, 4.4 × 10^4^, and 4.4 × 10^2^ CFU, respectively.

At 18 DOI, a 50 μL volume of the diluent was in ovo-injected alone (**DI**) or in combination with the PECV containing 1 of the 4 CFU doses of *E. coli*. These *E. coli* doses are subsequently described respectively as 10^2^, 10^4^, 10^6^, and 10^8^ CFU levels. A noninjected (**NI**) control group was also included. In the dispensing of diluent alone or in combination with PECV, injections penetrated the inner air cell membrane and targeted the AM. Eggs belonging to different treatment groups were kept separate and remained in the hatcher unit until they were injected. Treatments were injected using an Embrex Inovoject m injection machine (Zoetis, Durham, NC). Injection of diluent alone was administered first, followed by the consecutive administration of the lowest to highest *E. coli* doses. The injection process took a total of 1.5 h to complete. Cross-contamination prevention between treatments was as described by Lindsey et al. (2022). One egg from each tray (6 per treatment) was randomly selected to determine the accuracy of site of injection (Elliott et al., 2018) and embryonic developmental stage (Avakian, 2006). Once injection was complete, eggs (including the NI control) were returned to their same incubators for the hatcher phase and were placed in stacked hatching baskets with each basket divided in half by a divider, with each half of the basket containing equal numbers of eggs of the same treatment. All treatments were represented in each of the 6 total replicate blocks across the 3 incubators. In order to prevent possible contamination at hatch, the NI and DI treatment eggs were stacked respectively in the top 2 incubator levels, followed by the lowest to highest dose treatments being sequentially placed in the succeeding levels of the incubator.

### Hatchery Residue Analysis and Hatchling Evaluation at 22 DOI

According to the methods described by Lindsey et al. (2022), at hatch (22 DOI) no chicks were culled, and all hatchlings belonging to each treatment-replicate group were weighed and counted to determine mean hatchling BW and the percentage of viable chicks hatched from eggs that contained live embryos at the time of injection (18 DOI: **HI**). Embryo residue analysis included the percentages of contaminated eggs (**PCT**); mid-incubation (8–17 DOI, **PMD**) and late-incubation (18–19 DOI, **PLD**) embryonic deaths; embryos that were alive in the shell but had not attempted to pip (**PLNP**); embryos that were dead but that had pipped internally or externally (**PDP**); and live embryos that did not complete hatch but had pipped internally (**PLIP**) or externally (**PLEP**). Visible assessment of egg contamination (Peebles et al., 1998) and an analysis of hatched chicks at 22 DOI included determinations of hatchling mortality (free from the shell) and culled chicks (possessing dry, rough navels, or externalized intestines).

### Hatchling Quality Characteristics at 22 DOI

Female hatchlings were selected via feather sexing, and all females within a treatment (all 6 replicate groups) were pooled together. From 6 chicks in each pool, blood was collected via decapitation for serum ELISA analysis. In addition, within each pool of female hatchlings in each treatment group, 35 live female hatchlings per treatment were randomly selected for body length measurement. In an elongated position, body length was measured from the tip of the beak to the end of the extended third toe of the left leg (Joseph et al., 2006; Van den Brand et al., 2019). After body length measurement, the hatchlings were euthanized and the total BW and yolk sac weight of each of the 35 females per treatment were subsequently determined. Yolk sac dry matter and moisture contents were determined according to the procedures of Peebles et al. (1998). Yolk sac weight was expressed as a percentage of total BW, and yolk sac dry matter and moisture contents were expressed as percentages of total yolk sac weight. Using the total BW and yolk sac weight data, chick YFBW was calculated by subtracting yolk sac weight from total BW. Furthermore, BW-to-length ratio (**BWTLR**) and percentage body mass (**PBM**) were also calculated for each sampled female hatchling. The BWTLR was calculated by dividing the BW of the chick by its length. Mean PBM was calculated by dividing the yolk-free BW of the chick by its whole BW and multiplying the result by 100. Any remaining chicks (male and female) were euthanized.

### Serum ELISA Analysis

Indirect ELISA was used to determine serum anti-*E. coli* IgY levels in day-of-hatch birds. An overnight culture of *E. coli* (ATCC - 25922) was diluted 1:10 in ELISA coating buffer (Bio-Rad Laboratories, Hercules, CA) and used to coat 96-well plates by incubating overnight at 4.0°C. Following incubation, plates were washed 3 times with ELISA wash buffer (Bio-Rad Laboratories, Hercules, CA) and blocked overnight (4.0°C) with ELISA BSA Block (Bio-Rad Laboratories, Hercules, CA). Plates were washed twice with wash buffer, sealed, and stored at 4.0°C for no more than 2 wk prior to analysis. Serum samples were diluted 1:500 in blocking buffer, added to the prepared plate in triplicate, and incubated at room temperature for 3 h. Serum was removed by washing 4 times in wash buffer. Goat anti-Chicken IgG conjugated to HRP (Bio-Rad Laboratories, Hercules, CA) was diluted 1:10,000 in blocking buffer and incubated on the plate for 1 h and removed by washing the plate 4 times with wash buffer. Antibody signal was detected by adding TMB Core+ (Bio-Rad Laboratories, Hercules, CA) and incubating for 30 min followed by the addition of stop solution (2M H_2_SO_4_). Plates were read at 450 nm using an Epoch microplate spectrophotometer (Agilent Technologies, Santa Clara, CA). A standard curve was run using unlabeled chicken IgY (Southern Biotech, Birmingham, AL) and used to interpolate antibody concentrations.

### Statistical Analysis

A 1-way ANOVA was utilized to analyze the data. In the hatcher phase, a complete block design was utilized. Hatch basket section served as the experimental unit in the analyses of hatched chicks, residue eggs, and average hatchling BW. Individual female chick quality characteristics and serum ELISA and antibody concentration data were analyzed with individual bird representing the experimental unit. Least squares means were compared in the event of significant global effects. Global effects and least squares means differences were considered significant at *P* ≤ 0.05. All data were analyzed using the MIXED procedure of SAS software 9.4 (SAS Institute, 2013; Fatemi et al., 2022). Treatment means and LS means comparisons for variables that were not significantly affected by treatment are not described or discussed.

## RESULTS

### Egg Weight Loss, Site of Injection, and Embryo Staging

There were no significant differences between treatments for PEWL between 0 and 12 (*P* = 0.555) and between 12 and 18 (*P* = 0.975) DOI (Table 1). Of the 36 dye injections administered at 18 DOI, 31 (86.1%) were observed to be successfully administered in the AM. The rest of the injections were in the allantois (13.9%). The average embryonic developmental stage score at 18 DOI was 1.86, which was prior to a pipping response and prior to the positioning of the head of the embryo under its right wing.

**Table 1:** Effects of in ovo injection treatments on percentage egg weight loss (PEWL) between 0 and 12, and 0 and 18 d of incubation (DOI), hatchability of injected live embryonated eggs (HI), hatchling BW, and hatch residue variables at 22 DOI.

In ovo injection treatments^3^	PEWL 0–12	PEWL 12–18	PCT^1^	PLD^1^	PLNP 1	PDP^1^	PLIP^1^	PLEP^1^	Hatchling mortality^2^	HI	Hatchling BW
	%										g
NI	7.44	4.28	0^b^	1.40^b^	0.22	0.22	0^b^	0.63^c^	0.22	97.3^a^	39.0
DI	7.42	4.24	0^b^	4.26^a^	0	0.87	0^b^	0.67^c^	0	94.2^a^^b^	39.0
4.4 × 10^2^	7.32	4.3	0^b^	5.99^a^	0.22	0.87	0.87^a^^b^	3.05^bc^	0	89.0^bc^	39.0
4.4 × 10^4^	7.61	4.3	0.63^b^	4.16^a^	0.22	1.45	1.68^a^	2.97^b^^c^	0	88.9^bc^	38.8
4.4 × 10^6^	7.48	4.28	0.20^b^	5.14^a^	1.25	1.05	0.85^a^^b^	6.87^b^	0.20	84.4^c^	38.2
4.4 × 10^8^	7.48	4.29	2.06^a^	4.35^a^	1.58	1.50	2.23^a^	17.72^a^	0.42	71.2^d^	38.6
SEM	0.146	0.081	0.348	1.182	0.479	0.720	0.710	2.455	0.228	3.13	0.360
*P* value	0.555	0.975	<0.0001	0.017	0.156	0.528	0.024	<0.0001	0.373	<0.0001	0.191

a–d Means in the variable column with no common superscript differ significantly (*P* ≤ 0.05).

1 Percentages of contaminated eggs (PCT); late-incubation (18–19 DOI) embryonic deaths (PLD); embryos that were alive in the shell but had not attempted to pip (PLNP); embryos that were dead but that had pipped internally or externally (PDP); and live embryos that did not complete hatch but had pipped internally (PLIP) or externally (PLEP).

2 Hatchling mortality based on percentage of chicks observed dead in hatching basket sections.

3 NI = noninjected control treatment. DI = Marek's disease commercial diluent-injected control treatment. Poulvac vaccine *E. coli* CFU administered per egg are designated as: 4.4 × 10^2^, 4.4 × 10^4^, 4.4 × 10^6^, and 4.4 × 10^8^. *N* = 6 replicate hatching basket sections in each treatment, with each hatching basket section containing a maximum of 90 eggs (minus eggs removed for dye injection).

### Residue Eggs and Hatchability

Mean PMD (none observed), PLNP (*P* = 0.156), and PDP (*P* = 0.528) were not significantly affected by treatment. However, mean PCT (*P* < 0.0001) and PLD (*P* = 0.017) were significantly affected by treatment (Table 1). In the 10^8^ PECV dose treatment there was a significantly higher percentage of contaminated eggs when compared to all other treatments, with no significant differences between all the other treatments. Mean PLD was significantly lower in the NI treatment compared to all other treatments, with all other treatments being not significantly different from each other.

Mean PLIP (*P* = 0.024) and PLEP (*P* < 0.0001) were significantly affected by treatment (Table 1). In the 10^4^ and 10^8^ PECV dose treatments, there were significantly higher PLIP values than in the NI and DI treatments, with those in the 10^2^ and 10^6^ PECV dose treatments being intermediate. A significant higher PLEP was observed in the 10^8^ PECV dose treatment in comparison to all the other treatments. The PLEP in the 10^6^-dose treatment was also higher than that in the NI and DI controls, with that in the 10^2^ and 10^4^ PECV dose treatments being intermediate.

Mean HI was significantly (*P* < 0.0001) affected by treatment (Table 1). In the NI treatment, HI was significantly higher than all the other treatments except for the DI control group. A significantly higher HI was also observed in the DI treatment in comparison to that in the 10^6^ PECV dose treatment, with that in the 10^2^ and 10^4^ treatments being intermediate. Furthermore, HI in the 10^6^ PECV dose treatment was higher than that in the 10^8^ PECV dose treatment. There were no significant differences between the treatments for percentage hatchling mortality (*P* = 0.373) or for average hatchling BW (*P* = 0.191).

### Female Chick Quality Characteristics

Mean whole female chick BW was not significantly (*P* = 0.101) different between treatments. However, mean female YFBW was significantly (*P* = 0.034) affected by treatment (Table 2). Female YFBW in the DI and 10^4^ dose treatments was significantly higher than the YFBW in the 10^6^ and 10^8^ PECV dose treatments but was not significantly different from the NI and 10^2^ dose treatments. The NI and 10^2^ PECV treatments were intermediate to and not significantly different from any of the other treatment groups. Mean absolute (**YSW**) and percent (**PYSW**) yolk sac weight of the female chicks were also significantly (*P* < 0.0001) affected by treatment (Table 2). Those in the 10^8^ PECV dose treatment had a higher YSW in comparison to that in all the other treatment groups except for the 10^4^ treatment which was intermediate to the 10^8^ and 10^6^ treatments. The YSW in the 10^6^ treatment was greater than that in the NI and 10^2^ treatments with that in the DI treatment being intermediate. The PYSW value in the 10^8^ PECV dose treatment was greater than that in the NI and DI controls as well as the 10^2^ and 10^6^ PECV dose treatments. The PYSW values were also higher in the 10^4^-dose treatment in comparison to both control groups and the 10^2^ PECV treatment. Furthermore, the PYSW value in the 10^6^ PECV dose treatment was higher than that in the NI and 10^2^ dose treatments, with that in the DI control group being intermediate (Table 2).

**Table 2:** Effects of in ovo injection treatment on female hatchling body weight (BW) and hatchling quality characteristics at 22 d of incubation (DOI).

In ovo injection treatments^2^	BW	YFBW^1^	YSW^1^	DYSW^1^	PYSW^1^	PYSM^1^	PDYSW^1^	PBM^1^	Body length	BWTLR^1^
	g	g	g	g	%				cm	g/cm
NI	37.8	35.9abc	3.74^c^	1.85^c^	9.69^d^	50.7abc	49.3abc	95.2^a^	18.36^a^	2.06^c^
DI	38.8	36.7^a^	4.19^bc^	2.11^bc^	10.77^cd^	50.4^bc^	49.7^a^^b^	94.7^a^^b^	18.38^a^	2.11^bc^
4.4 × 10^2^	37.4	35.6abc	3.73^c^	1.81^c^	9.83^d^	51.7^a^	48.3^c^	95.2^a^	18.23^a^	2.05^c^
4.4 × 10^4^	38.6	36.2^a^^b^	5.01^a^^b^	2.45^a^^b^	12.85^a^^b^	51.3^a^^b^	48.7^b^^c^	93.7^cd^	17.52^b^	2.21^a^
4.4 × 10^6^	37.0	34.8^c^	4.49^b^	2.21^b^	11.97^b^^c^	51.3^a^^b^	48.7^b^^c^	94.1^b^^c^	17.56^b^	2.09^bc^
4.4 × 10^8^	37.8	35.1^c^	5.32^a^	2.69^a^	14.01^a^	49.9^c^	50.2^a^	92.9^d^	17.73^b^	2.15^a^^b^
SEM	0.72	0.63	0.326	0.175	0.757	0.641	4.16	0.42	0.122	0.042
*P* value	0.101	**0.034**	**<0.0001**	**<0.0001**	**<0.0001**	**0.040**	**<0.0001**	**<0.0001**	**<0.0001**	**0.002**

a–d Means in the variable column with no common superscript differ significantly (*P* ≤ 0.05).

1 Yolk-free BW (YFBW; BW-YSW), absolute yolk sac weight (YSW), absolute dry yolk sac weight (DYSW), percent yolk sac weight [PYSW; (YSW/BW) × 100], percent yolk sac moisture [PYSM; ((YSW − DYSW)/YSW) × 100], percent dry yolk sac weight [PDYSW; (DYSW/YSW) × 100], percent body mass [PBM; (YFBW/BW) × 100], and body weight-to-length ratio (BWTLR; BW/body length).

2 NI = noninjected control treatment. DI = Marek's disease commercial diluent-injected control treatment. Poulvac vaccine *E. coli* CFU administered per egg is designated as: 4.4 × 10^2^, 4.4 × 10^4^, 4.4 × 10^6^, and 4.4 × 10^8^. *N* = 35 individual chicks from a pool of 6 replicate hatching basket sections in each treatment.

Mean absolute (**DYSW**) and percentage (**PDYSW**) dry yolk sac weight of female chicks were significantly (*P* < 0.0001) affected by treatment (Table 2). Mean DYSW in the 10^8^ PECV dose treatment was significantly higher than that in all the other treatments, except for that in the 10^4^ PECV dose treatment which was intermediate to the 10^8^ and 10^6^ dose treatments. The DYSW of birds in the 10^4^ and 10^6^ PECV dose treatments was higher than those in the NI and 10^2^ PECV dose treatments, with that in the DI control treatment being intermediate. The PDYSW of birds in the 10^8^ PECV dose treatment was significantly higher than that in all the other treatments, except for the NI and DI control groups. The PDYSW of those in the DI treatment was higher than those in the 10^2^ PECV dose treatment, with that in the 10^4^ and 10^6^ PECV dose treatment groups being intermediate. The PDYSW of the NI control group was intermediate to and not significantly different from any of the other treatment groups. Mean percent yolk sac moisture (**PYSM**) in female chicks was also significantly (*P* = 0.040) affected by treatment (Table 2). The PYSM of the female chicks was significantly higher in the 10^2^ PECV dose treatment in comparison to the DI and 10^8^ PECV dose treatments. The PYSM in the 10^4^ and 10^6^ PECV dose treatments were also higher than that in the 10^8^ PECV dose treatment, whereas the NI and DI groups were intermediate. In addition, the NI control group was intermediate to and not significantly different from any of the treatments (Table 2).

Mean PBM of female chicks was significantly (*P* < 0.0001) affected by treatment (Table 2). Birds in the NI and 10^2^ PECV dose treatments had a significantly higher PBM than those in the 10^6^ PECV dose treatment, with those in the DI treatment being intermediate. Birds in the 10^6^-dose treatment had a higher PBM than those in the 10^8^ PECV dose treatment, with those in the 10^4^-dose treatment being intermediate (Table 2). Mean body length of female chicks was significantly (*P* < 0.0001) different between treatments (Table 2). The birds in the NI, DI, and 10^2^ PECV dose treatments had significantly longer body lengths compared to those in the 10^4^, 10^6^, and 10^8^ PECV dose treatments. Body weight to length ratio (**BWTLR**) of female chicks was also significantly (*P* = 0.002) different between treatments (Table 2). The BWTLR of birds in the 10^4^-dose treatment was significantly higher than that in all the other treatments except for that in the 10^8^-dose treatment. The BWTLR in the 10^8^ PECV dose treatment was higher than that in the NI and 10^2^ PECV dose treatments, within that in the DI and 10^6^ dose treatments being intermediate (Table 2).

### Female Chick Serum IgY Response at Hatch

**Figure 1:**
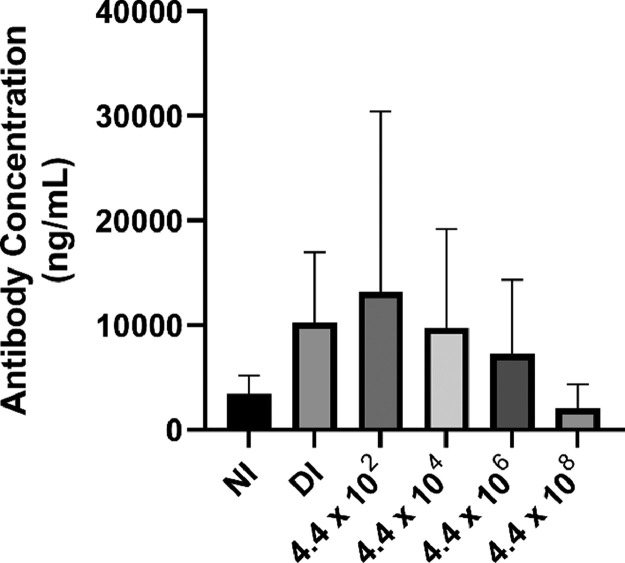
Effect of in ovo injection on circulating IgY at hatch. NI = noninjected control treatment. DI = Marek's disease commercial diluent-injected control treatment. Poulvac vaccine *E. coli* CFU administered per egg are designated as: 4.4 × 10^2^, 4.4 × 10^4^, 4.4 × 10^6^, and 4.4 × 10^8^. Serum was collected from 6 birds per treatment immediately following hatch. Circulating IgY was measured using an indirect ELISA and quantified against a chicken IgY standard curve. No significant difference in antibody titer was found among treatments (*P* = 0.675).

Most of the sampled female chicks were found to be positive for serum IgY against *E. coli* at hatch (Table 3). Analysis of the antibody concentration of the positive samples showed no significant difference between the treatments (*P* = 0.675; Figure 1).

**Table 3:** Percentage of ELISA positive chicks at hatch by treatment.

In ovo injection treatments^1^	Number of female chicks sampled^2^	Number of female chicks testing positive	Percentage positive chicks
NI	6	3	50
DI	6	2	33
4.4 × 10^2^	6	4	67
4.4 × 10^4^	6	4	67
4.4 × 10^6^	6	5	83
4.4 × 10^8^	6	3	50

1 NI = noninjected control treatment. DI = Marek's disease commercial diluent-injected control treatment. Poulvac vaccine *E. coli* CFU administered per egg is designated as: 4.4 × 10^2^, 4.4 × 10^4^, 4.4 × 10^6^, and 4.4 × 10^8^.

2 Female chicks were randomly selected from each treatment.

## DISCUSSION

Across the 6 tray levels and the 3 incubators, there were no significant differences between treatments for PEWL between both the 0 to 12 and 12 to 18 DOI periods, indicating that the eggs in all the treatment groups experienced similar incubational conditions, thereby ensuring that the noted effects of treatment on the variables examined were not confounded by environmental influences during incubation. Moreover, with only 6 tray levels in each incubator, a possible stratification effect in each incubator remained minimal.

A comparison of the pre- and postplating results in which 8.8 × 10^9^ and 3.4 × 10^9^ CFU/mL, respectively, were observed, indicates that a negligible number of bacteria were lost during the injection process, and that an appreciable number of bacteria would have been administered within the treatment groups. The efficacy of the administered PECV is further supported by the embryo developmental stage and site of injection results. In the current study, 86.1% of the injections were successfully administered in the AM. The mean embryo stage score at 18 DOI of 1.86 was like the 2.09 score reported by Sokale et al. (2018) for Ross 708 broiler embryos at 18.5 DOI. It was also like the 1.65 score reported by Elliott et al. (2022) for layer embryos at 18 DOI. According to Avakian (2006), embryos injected at 18 DOI with an Inovoject machine have a greater than 90% chance of their injections occurring in the AM, with approximately 5.79% of their injections occurring in the allantoic sac, air cell, or yolk sac. The current results of this and previous studies indicate that most of the injections administered at 18 DOI were successfully administered in the AM. Furthermore, employing the same amniotic injection procedure as in the current study and using PCR results from swabs of the amnion membrane at 19 DOI, Lindsey et al. (2022) reported that all diluent-injected control eggs were negative for Poulvac *E. coli* presence, whereas eggs injected with 6.5 × 10^1^ or 6.5 × 10^2^ CFU were 50% positive and those injected with 6.5 × 10^3^ or 6.5 × 10^4^ CFU were 100% positive for *E. coli* presence.

Like the decrease in HI and increase in PLIP and PLEP in response to the increased dosage of the in ovo-injected PECV in this study, Lindsey et al. (2022) reported that HI decreased, and embryonic mortalities increased in response to the in ovo injection of 6.5 × 10^4^ CFU of the PECV into the AM. These results are also like those of Elliott et al. (2017), who investigated the injection of the F-strain of *Mycoplasma gallisepticum* in Hy-Line W-36-layer embryos at 18 DOI. In that study, in which 5.5 × 10^6^, 5.5 × 10^4^, 5.5 × 10^2^, and 5.5 CFU concentrations of the Poulvac Myco F vaccine were used, it was found that HI decreased as dose increased, with an approximate 60% HI observed in the full dose treatment. More specifically, there was a significantly higher percentage of embryos that died while pipping after receiving a 1 × 10^4^ or 1 × 10^6^ CFU dose of the Poulvac Myco F vaccine. These combined results indicate that when injected at 18 DOI into the AM, the *E. coli* of the PECV at the dosages delivered in this study, particularly between 10^4^ and 10^8^ CFU, compromised late embryonic viability, thereby preventing the ability of the embryo to complete a normal hatching process. The increases in PLIP and PLEP were evidently the major cause of the decrease in HI. It was also noted that the highest percentages of PLIP and PLEP corresponded with the highest percentages of PDP in the 10^8^ CFU treatment, suggesting that given a longer observational period after 22 DOI, the PLIP and PLEP embryos would eventually die. A possible reason for the dose-dependent effects of the in ovo injection of the PECV could be linked to an increase in *E. coli* infection after the injection of the 10^8^ CFU level of the PECV. Furthermore, the current results showed that in comparison to all the other treatments, higher percentages of contaminated eggs were observed when a full dose of the PECV was injected.

Maternal antibodies, transferred from the breeder hen to the embryo via the egg yolk, are a chick's leading defense against pathogens until they can produce their own immune cells (Hincke et al., 2019). The hens that laid the hatching eggs used in the current study were vaccinated for *E. coli* according to company recommendations (Hy-Line International, 2020), and most of the resulting progeny were positive for serum IgY against *E. coli* at hatch as expected (Malkinson, 1965; Heller et al., 1990). There were, however, no differences in antibody concentration among the positive samples regardless of if the embryos had been administered the *E. coli* vaccine in ovo or not. These findings hint that the maternal antibodies may not have interfered with or neutralized the Poulvac *E. coli* vaccine strain within the embryonated egg (Negash et al., 2004). Additionally, the levels of antibodies that they possessed, if they acted to neutralize the vaccine strain of *E. coli*, may not have been sufficient to protect them from the large amounts of *E. coli* administered at 18 DOI. In a study conducted by Weber and Rodenberg (2019), 1-day-old specific-pathogen-free chicks were vaccinated with PECV by coarse spray and then challenged with APEC at either 7, 14, or 21 d of age (**DOA**). While control chicks showed a high percentage of colibacillosis lesions at 7, 14, and 21 d of growout, chicks that had been vaccinated with the PECV had a significantly lower presence of lesions at 14 and 21 d of growout. Blood samples were taken on day of hatch to test for the presence of maternal antibodies in chicks that later served as unvaccinated controls or that were vaccinated at 1 d of age with the PECV. While it was found that the specific-pathogen-free chicks did indeed carry maternal antibodies for *E. coli*, it was also concluded that the presence of maternal antibodies alone were not sufficient in protecting chicks from an APEC infection.

Another basis for a defective hatching process and increased rate of embryonic mortality could be due to the immaturity of the immune systems of the embryos. It is also well documented that the immunity of chickens is age-related (van Ginkel et al., 2015). By 18 DOI, chicken embryos can activate an immune response against pathogens that they come into contact with (Hincke et al., 2019 ). However, because immune competence tends to increase with age, older birds tend to have a better immune response to pathogens than embryos or young chicks (Alkie et al., 2019). Furthermore, an increase in mortality is not seen when the PECV is administered to chickens via coarse spray or drinking water at 1 d of age (Zoetis, 2021a), or to pullets via drinking water at 5 d of age, or at 14 and 30 wk of age (Christensen and Nielsen, 2020). The adaptive immunity of the gut-associated lymphoid tissue matures toward the end of the second week of life (Bar-Shira and Friedman, 2006). Therefore, it is likely that only an innate immune response was activated when the embryos were vaccinated at 18 DOI in the current study, and that the response was not strong enough to overcome the large amounts of *E. coli* deposited in the AM. This indicates that chicken embryos are more prone to enteric pathogens such as *E. coli* due to immaturity of the adaptive immune response.

Triplett et al. (2018) administered 3 different probiotic strains of bacteria (*Lactobacillus acidophilus, Bacillus subtilis*, and *Bifidobacterium animalis*) by in ovo injection to broiler hatching eggs at 18 DOI. In the *Bacillus subtilis* treatment, there was a significant increase in the percentage of embryos that died during the pipping process. Upon consideration of various possible reasons for the increase in the number of embryos that died while pipping, Triplett et al. (2018) suggested that the embryos in that treatment group may have lacked sufficient energy reserves to immunologically resist the bacterial challenge. This same type of response to the *E. coli* bacterial challenge, involving insufficient energy reserves as well as an immature immunity, may also have occurred in the current study.

The results of the determinations of the various subsequent quality characteristics of the hatchlings in response to the imposed treatments revealed that the adverse posthatch effects of the higher in ovo PECV dosages on the embryo carried over into the posthatch period. In comparison to the DI control, female chick YFBW and body length were decreased in the 10^6^ and 10^8^ PECV dose treatments, and those in the 10^8^-dose treatment had a lower PBM. Chick body length has been viewed as a variable that can be a predictor of later chick quality, with a longer length being associated with improved posthatch performance (Molenaar et al., 2008). Although compensatory growth cannot be precluded, a higher dose of the PECV may lead to lower chick quality in the growout phase. It is well documented that metabolic enzyme activity increases activity between 17 and 20 DOI (Rinaudo et al., 1979). Their activities then become low in the early posthatch phase (Moriuchi and Deluca, 1974). Additionally, the development of tissues linked to humoral immunity (spleen and bursa of Fabricius) and cell-mediated immunity (thymus) occur by 19 DOI (Moriuchi and Deluca, 1974). Furthermore, small intestine development continues through the first wk of posthatch life (Uni et al., 1995a,b; Applegate et al., 1999; Geyra et al., 2001). It is worth mentioning that during the fast-growing stage of embryogenesis (the last 3–4 DOI), the embryo requires sufficient nutrients to support a proper hatch as well as hatchling quality. Additionally, this stage of embryogenesis is highly associated with posthatch performance (Peebles, 2018). Not only may in ovo injection of the PECV negatively affect embryo development and result in a delay in the proper development of immune-related organs, but it may also further lead to changes in the morphology of the small intestine, which is linked to lower nutrient absorption (Onderci et al., 2006). However, further research is needed to determine histomorphological changes in the small intestine and immune-related organs that may occur in response to the in ovo injection of the PECV.

In comparison to both controls, YSW, PYSW, and DYSW were significantly higher in the 10^8^ PECV dose treatment. This indicates that the hatched chicks from the higher PECV treatment group retained heavier yolk sacs. Having a larger residual yolk sac could be another result of a compromised physiology of the embryos in response to the injection of the PECV. A mounted immunological resistance to the bacterial insult could have led to a delay in development, thus causing the affected chicks to retain more yolk. These results, however, differ from those of Elliott et al. (2017), who found no significant differences in YFBW, YSW, PYSW, DYSW, or PDYSW between controls and embryos that were in ovo-vaccinated with the Poulvac Myco F vaccine at 18 DOI. Nevertheless, the results of the current study were like the results reported in a study by Montgomery et al. (1999), who injected Ec^NAL^, an adapted form of *E. coli*, into the chorioallantoic sac of broiler embryos at 12 DOI. Of the chicks that hatched, it was discovered that the chicks injected with Ec^NAL^ had heavier yolks and a higher yolk mass relative to body mass when compared to control groups. When birds were reared until 21 DOA, it was further found that the mean BW of the chicks that had been injected with Ec^NAL^ were significantly lower than the mean BW of the control groups (Montgomery et al., 1999).

In conclusion, it was shown in this study that the PECV can be successfully delivered to the AM of Hy-Line W-36-layer hatching eggs at 18 DOI. Nevertheless, the injection of the PECV into the AM at 18 DOI at the doses administered in this study may incur detrimental effects on late embryo livability, subsequent HI, and hatchling quality characteristics. Furthermore, in ovo injection of the vaccine to embryos rather than by spray or drinking water to hatchlings, as is currently recommended, appears to have an overwhelming effect on the newly forming immune system of the embryo, yielding potential undesired results. Further exploration into the growout period to examine possible compensatory growth should be considered.
